# Optimizing crop quality and yield: Assessing the impact of integrated potassium management on Chinese cabbage (*Brassica rapa* L. subsp. *chinensis*)

**DOI:** 10.1016/j.heliyon.2024.e36208

**Published:** 2024-08-16

**Authors:** Mahendra Choudhary, Sourabh Kumar, Santosh Onte, Vijendra Kumar Meena, Dhruba Malakar, Kamal Garg, Sanjeev Kumar, Mahendra Vikram Singh Rajawat, Mukesh Kumar Awasthi, Balendu Shekher Giri, Durgesh Kumar Jaiswal, Shiva Dhar, Elisa Azura Azman, Sanjivkumar Angadrao Kochewad

**Affiliations:** a Department of Agronomy, College of Agriculture, G. B. Pant University of Agriculture and Technology, Pantnagar, Udham Singh Nagar, Uttarakhand, 263145, India; b Veer Kunwar Singh College of Agriculture, Dumaraon, Buxar, 802136, Bihar, India; c Centre for Water Resources Development and Management (CWRDM), Calicut, 673571, India; d ICAR-National Dairy Research Institute, Karnal, Haryana, India; e Dhanuka Agritech Limited, Dhanuka Agritech Research and Technology Center, Palwal-Aligargh Road, Sihol, 121102, Haryana, India; f College of Natural Resources and Environment, Northwest A&F University, Taicheng Road 3 Yangling, Shaanxi, 712100, China; g Sustainability Cluster, University of Petroleum and Energy Studies (UPES), Dehradun, Uttarakhand, 248007, India; h Department of Biotechnology, Graphic Era (Deemed to be University), Dehradun, 248002, Uttarakhand, India; i ICAR-Indian Agricultural Research Institute, New Delhi, 110012, India; j Faculty of Agriculture, Department of Crop Science, Universiti Putra Malaysia, Serdang, 43400, Malaysia; k ICAR-National Institute of Abiotic Stress Management, Baramati, 413115Maharashtra, India

**Keywords:** Farmyard manure, Feed quality, Nano-potash, Plant growth promoting rhizobacteria, Proximate composition, Yield

## Abstract

Potassium, a pivotal macronutrient essential for growth, development, and crop yield, serves as a critical determinant of soil productivity. Its depletion disrupts the equilibrium of soil nutrients, prompting an investigation into integrated potassium management strategies to address this challenge. A field experiment was conducted during the winter season of 2020 using a randomized complete block design, with eight treatments, each replicated three times in Chinese cabbage (*Brassica rapa* L. subsp. *chinensis*). These treatments comprised standard (100 %) and reduced (75 % and 50 %) rates of the recommended dose of potassium (RDK) via muriate of potash (MOP). Variations in the inclusion and exclusion of plant growth-promoting rhizobacteria (PGPR), farmyard manure (FYM) as 25 % of the potassium recommendation, and foliar spray of nano potash were systematically implemented. Findings unequivocally demonstrated that the treatmentT_8_, involving 100 % RDK +25 % K through FYM + PGPR + nano K fertilizer spray at 25 and 40 DAS, yielded significant improvements in both green fodder (64.0 t ha^−1^) and dry fodder (7.87 t ha^−1^).Moreover, T_8_ exhibited the highest values for total ash (8.75 %), total ash yield (68.9 ± 2.88 kg ha^−1^), ether extract (2.85 %), ether extract yield (22.4 ± 0.88 kg ha^−1^), crude protein (9.71 %), and total crude protein yield (76.4 ± 3.21 kg ha^−1^). Conversely, a marked reduction was observed in various fiber components and carbohydrate fractions upon application of the T_8_ treatment. The lowest values of yield, crude protein content, total ash ether extract were recorded in treatment T_1_ (control) applied with no potassium. This investigation underscores the inadequacy of the recommended potassium dose in achieving optimal productivity, necessitating a re-evaluation of potassium fertilization levels. The integrated approach involving FYM, PGPR, and nano potash, coupled with the recommended potassium dose through MOP, emerges as a promising avenue for augmenting both yield and quality parameters in Chinese cabbage.

## Highlights

• Combined use of FYM, PGPR, and nano K, alongside 75 % RDK emerges as promising approach.
• Integrated K management significantly improve yield, proximate composition, and feed quality.
• It contributed to reduce the fiber fractions in Chinese cabbage.
• It contributed to improve fodder quality and profitability for livestock farmers.

## Graphical abstract

**Figure:**
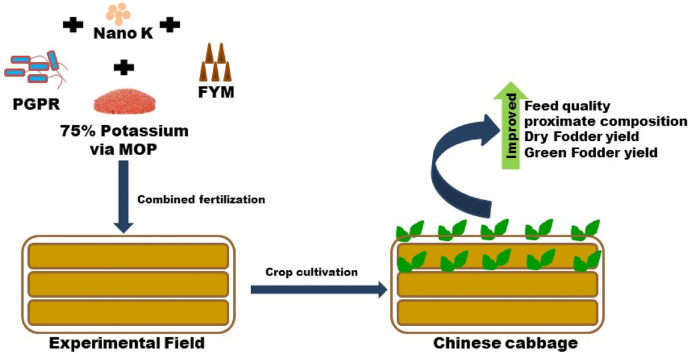

## 1 Introduction

Livestock plays a vital role in economy by employment generation as well as providing supplementary family income in the countries with small and marginal farmers. Livestock performance is mainly dependent on its feeding. Forages are the mainstay of animal wealth. The profitability of livestock production is directly dependent on the sources of feed and fodder; hence, the quality of fodder can play an important role in increasing the animal productivity [1,2]. However, the unavailability of quality fodder to meet the nutritional requirement remains a significant barrier, contributing to lower productivity and profitability in livestock sector.

Introducing crops that offer both high-quality and abundant fodder during lean periods can significantly enhance livestock health and productivity [3,4]. Chinese cabbage (*Brassica rapa* L. subsp. *chinensis*) emerges as a promising solution within the *Brassicaceae* family, not only providing nutritious fodder but also edible oil, making it versatile for North India agriculture. Its rapid growth cycle (75–80 days) positions Chinese cabbage as an ideal option for ensuring consistent green fodder supply during periods of scarcity. However, achieving ideal fodder quality and quantity hinges on factors such as optimal sowing time, effective weed control [5], and strategic fertilizer application [6].

While nitrogenous and phosphatic fertilizers are commonly used in nutrient management, potassium (K) often receives inadequate attention due to misconception that Indian soils are inherently rich in this nutrient. Patra et al. [7] reported that soil K data from eight districts in different regions of India and showed that only two districts fall in the high fertility class, while the rest are in the low to medium categories. These discrepancies are largely due to intensive cropping systems and low potassium application. This oversight contributes to inferior fodder quality and reduced yield, exacerbating potassium depletion in soil. Furthermore, the cultivation of high-yielding varieties intensifies potassium depletion by increasing nutrient uptake from natural reservoirs [8].

Potassium is known as a quality element that plays a major role to improves root growth, regulates osmosis, maintain ionic balance, and improves water retention thereby preventing wilting and energy loss [9]. It plays a pivotal role in nutrient assimilation, protein synthesis, cellulose formation and disease resistance, particularly crucial in oilseed crops for enhancing fatty acid biosynthesis and oil quality [10]. The optimal range of potassium for most vegetable crops, including Chinese cabbage, is considered to be between 200 and 300 ppm (parts per million) in the soil [[11], [12], [13]]. Despite its importance, traditional sources like muriate of potash are not always economically viable for farmers.

Alternative sources of K such as farmyard manure (FYM), plant growth promoting rhizobacteria (PGPR) and nano potash present a viable solution. FYM improves soil condition and fertility, while PGPR enhances productivity by mobilizing nutrients bound in organic matter when combined with organic amendments [14,15]. Nano-potash has shown effectiveness in enhancing growth parameters, yield and quality in various crops [16]. Integrated approaches combining FYM, PGPR, and nano-potash have demonstrated significant improvements in plant health, yield, and soil quality [16,17].

Based on the above information, we hypothesize that integrated K management, incorporating sources such as FYM, PGPR, and nano-potash, will significantly improve the yield, quality, and nutritional value of Chinese cabbage (*Brassica rapa* L. subsp. *chinensis*) compared to traditional potassium fertilization methods.

Given the critical role of K in enhancing crop productivity and soil health, this study aims to evaluate the impact of integrated K management on Chinese cabbage (*Brassica rapa* L. subsp. *chinensis*). The management methods include FYM, PGPR, and nano-potash, along with different levels of K through MOP. We aim to assess yield, proximate composition, fiber fraction, and feed quality. The novelty of this work lies in the integrated approach to K management, combining traditional and innovative sources like FYM, PGPR, and nano-potash to optimize nutrient uptake and improve crop yield and quality. Further, by addressing these objectives, the research seeks to fill gaps in current knowledge regarding optimal K utilization in agriculture, thereby supporting enhanced livestock nutrition and sustainable agricultural practices.

## 2 Material and methods

### 2.1 Experimental details

The investigation transpired at the experimental research farm situated in the Agronomy Section of the ICAR-National Dairy Research Institute, Karnal, Haryana, during the winter season of 2020. Positioned within the Trans Indo-Gangetic Plain of India at coordinates 29.45° N latitude, 76.58° E longitude, and an elevation of 245 m above mean sea level, this region is characterized by sub-tropical climate. The climate exhibits dry-hot conditions during summer and cold climate during winter. The rainfall in this locale is received from both the North-East monsoon in winter and the South-West monsoon in the rainy season. In accordance with the meteorological data for the year 2020, the recorded rainfall from October to December totaled 43.6 mm (Suppl. Fig. 1). The 46th standard week (12th November −18th November) witnessed the maximum average relative humidity (98.3 %), while the 40th standard week during the crop period experienced the highest evaporation rate (4.8 mm day^−1^), maximum temperature (34.5 °C), and sunshine hours (8.6 h day^−1^). The soil composition of the experimental field is characterized as clay loam, exhibiting elevated levels of organic carbon (0.66 %), a neutral pH (7.46), low nitrogen content (198 kg ha^−1^), high phosphorous content (29.1 kg ha^−1^), and medium potassium availability (235 kg ha^−1^).

The experimental design employed in this study was a randomized complete block design (RCBD), consisting of eight treatment combinations (Table 1). The treatments encompassed the following categorizations: T_1_ - control (No K); T_2_ - recommended dose of fertilizer (RDK) via muriate of potash (MOP); T_3_ - 75 % RDK (MOP) + nano K fertilizer spray at 25 and 40 days after sowing (DAS); T_4_ - 50 % RDK + PGPR + nano K fertilizer spray at 25 and 40 DAS; T_5_ - 75 % RDK + PGPR + nano K fertilizer spray at 25 and 40 DAS; T_6_ - 50 % RDK+25 % K infusion through FYM + PGPR + nano K fertilizer spray at 25 and 40 DAS; T_7_ - 75 % RDK + 25 % K enrichment through FYM + PGPR + nano K fertilizer spray at 25 and 40 DAS; T_8_ - 100 % RDK + 25 % K augmentation through FYM + PGPR and nano K fertilizer spray at 25 and 40 DAS. The seedbed preparation adhered to the specific requirements of the crop, executed using a cultivator and leveller with a subtle gradient to facilitate optimal irrigation. The chosen fodder mustard variety, "Chinese cabbage," was sown at a seed rate of 5 kg ha^−1^ with a spacing of 30 cm × 10 cm. Fertilizer application was meticulously carried out at a rate of 120 kg N ha^−1^ and 60 kg P_2_O_5_ ha^−1^. Of this, half of the nitrogen and the full amount of phosphorus were applied as the basal dose, with the remaining nitrogen administered at 30 DAS. Potassium supplementation was accomplished through muriate of potash, farmyard manure as the basal dose, and a foliar application of nano potash at 0.3 % at 25 and 40 DAS, in accordance with the stipulations outlined in Table 1. To incorporate 25 % of the recommended fertilizer dose through farmyard manure, FYM analysis was conducted on a dry weight basis. The requisite amount of FYM for 25 % potassium application was then calculated. To maintain a balanced dose of nitrogen and phosphorus, the quantity provided through FYM was also determined and subtracted from the overall fertilizer dose. Furthermore, the seeds underwent inoculation with plant growth-promoting rhizobacteria, following the specified treatment details. The cultivation of Chinese cabbage (*Brassica rapa* L. subsp. *chinensis*) involved three replications for each treatment.

**Table 1:** Treatment description of integrated potassium management in the experimental conditions.

Treatment Symbol	Treatment Details
T_1_	Control (No K application)
T_2_	RDK (MOP)
T_3_	75%RDK (MOP) + foliar spray of nano potash at 25 and 40 DAS
T_4_	50 % RDK + PGPR + foliar spray of nano potash at 25 and 40 DAS
T_5_	75 % RDK + PGPR + foliar spray of nano potash at 25 and 40 DAS
T_6_	50%RDK +25 % K through FYM + PGPR + foliar spray of nano potash at 25 and 40 DAS
T_7_	75%RDK +25 % K through FYM + PGPR + foliar spray of nano potash at 25 and 40 DAS
T_8_	100%RDK +25 % K through FYM + PGPR + foliar spray of nano potash at 25 and 40 DAS

*RDK= Recommended dose of potassium; PGPR=Plant Growth Promoting Rhizobacteria; FYM= Farmyard manure.

### 2.2 Estimation of yield and proximate composition of Chinese cabbage

Chinese cabbage underwent manual harvesting, with the recording of fresh weight and subsequent conversion of yield (kg plot^−1^) into tons per hectare (t ha^−1^). Representative samples were systematically extracted from each experimental plot and subjected to a 48-h desiccation period in a hot air oven maintained at 60 °C. The resulting dry fodder yield was then computed in tons per hectare. Subsequent to desiccation, the samples underwent crushing in a Wiley mill and were sieved at a 1 mm aperture, followed by preservation in sealed polythene bags for subsequent analytical procedures. The analysis of the fodder's proximate composition, encompassing crude protein (CP), ether extract (EE), and total ash (TA), was conducted utilizing the association of official analytical chemists (AOAC) method [18]. The calculation of crude protein involved the multiplication of the nitrogen content, determined through the Kjeldhal method, by a factor of 6.25. Subsequently, the values for crude protein, ether extract, and total ash content were further multiplied by the dry fodder yield, resulting in the derivation of crude protein yield, ether extract yield, and total ash yield on a per-hectare basis.

### 2.3 Estimation of fiber and carbohydrate fractions of Chinese cabbage

The methodology employed for assessing neutral detergent fiber (NDF), acid detergent fiber (ADF), and acid detergent lignin (ADL) adhered to the protocols outlined by Van Soest, Robertson and Lewis [19]. Acid insoluble ash (AIA) was determined from the acid detergent fiber using the methodology described by Oke [20]. Neutral detergent insoluble nitrogen (NDIN) and acid detergent insoluble nitrogen (ADIN) were quantified by analyzing residues from NDF and ADF, respectively, employing the Kjeldahl Nitrogen estimation process as outlined by Ref. [21]. The values for neutral detergent insoluble crude protein (NDICP) and acid detergent insoluble crude protein (ADICP), expressed as a percentage of dry matter (DM), were computed by multiplying the NDIN and ADIN values by a factor of 6.25. To represent NDICP and ADICP on a percentage of crude protein (CP) basis, the values initially calculated on a percentage of dry matter basis were subsequently divided by the CP content of the sample. Total carbohydrate (%), structural carbohydrate (%), non-structural carbohydrate (%), cellulose (%), and hemicellulose (%) were estimated in accordance with the procedures delineated by Van Soest, Robertson and Lewis [19].

### 2.4 Feed quality estimation of Chinese cabbage

The nutritive characteristics of the feed, specifically dry matter intake (DMI), dry matter digestibility (DMD), total digestible nutrients (TDN), relative feed value (RFV), and relative forage quality (RFQ), were assessed employing the methodologies outlined by Horrocks and Valentine [22] and Undersander, Moore and Schneider [23]. The computation of these parameters followed established formulae as prescribed by these authors.

(1) $$\text{DMI}\;\left(\%\;\right)=\frac{120}{\text{NDF}\%}$$

(2) DMD(%)=88.9−(0.779xADF%)

(3) TDN(%)=(‐1.29xADF%)+101.35

(4) $$\text{RFV}=\frac{\text{DMI}\;\left(\%\right)\;x\;\text{DMD}\;\left(\%\right)}{1.29}$$

(5) $$\text{RFQ}=\frac{\text{DMI}\;\left(\%\right)\;x\;\text{TDN}\;\left(\%\right)}{1.23}$$

### 2.5 Statistical analysis

The data obtained from the field experiment underwent statistical analysis utilizing R-software version 4.1.2 at a significance level of 5 % (*p* ≤ 0.05). The analysis of variance (ANOVA) test was employed to assess and compare the means, following the methodology outlined by Gomez and Gomez [24]. GraphPad PRISM 8.0 facilitated the creation of graphical representations, while correlation analysis was conducted using JASP version 0.18.3.0.

## 3 Results

### 3.1 Green and dry fodder yield

**Fig. 1:**
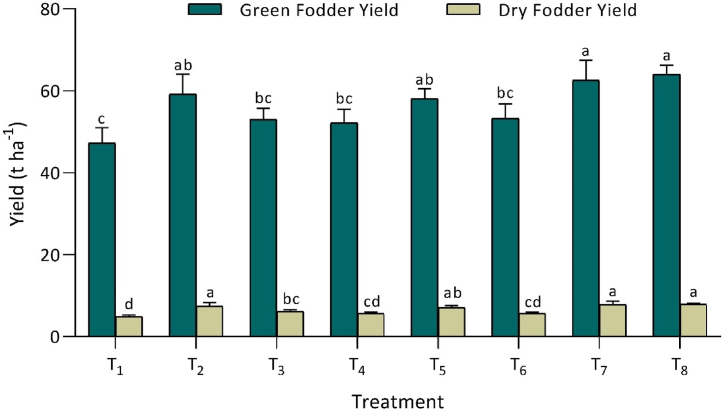
T_1_ - control (No K); T_2_ - recommended dose of potassium fertilizer (RDK) via muriate of potash (MOP); T_3_ - 75 % RDK (MOP) + nano K fertilizer spray at 25 and 40 days after sowing (DAS); T_4_ - 50 % RDK + PGPR + nano K fertilizer spray at 25 and 40 DAS; T_5_ - 75 % RDK + PGPR + nano K fertilizer spray at 25 and 40 DAS; T_6_ - 50 % RDK+25 % K infusion through FYM + PGPR + nano K fertilizer spray at 25 and 40 DAS; T_7_ - 75 % RDK + 25 % K enrichment through FYM + PGPR + nano K fertilizer spray at 25 and 40 DAS; T_8_ - 100 % RDK + 25 % K augmentation through FYM + PGPR and nano K fertilizer spray at 25 and 40 DAS. Fig. 1. Effect of integrated potassium management on yields of Chinese cabbage.

The green and dry fodder yield of Chinese cabbage (*Brassica rapa* L. subsp. *chinensis*) exhibited significant variations as a consequence of integrated K application (Fig. 1). Among the treatments, T_8_ recorded the highest green yield (64.0 ± 2.2 t ha^−1^) and dry fodder yield (7.87 ± 0.33 t ha^−1^). In contrast, the control treatment (T_1_) exhibited the lowest green (47.3 ± 3.7 t ha^−1^) and dry fodder yield (4.88 ± 0.44 t ha^−1^). Treatment T_8_ demonstrated comparable results with T_7_, T_2_, and T_5_. Additionally, T_1_ was statistically similar to T_3_, T_4_, and T_6_ for green fodder yield, while being equivalent to T_4_ and T_6_ for dry fodder yield. Notably, the integrated K application, particularly 100 % RDK through MOP alone, significantly enhanced the yield of Chinese cabbage in this experiment. This showed that integrated K application significantly enhanced Chinese cabbage green and dry fodder yields compared to control.

### 3.2 Proximate composition

**Fig. 2:**
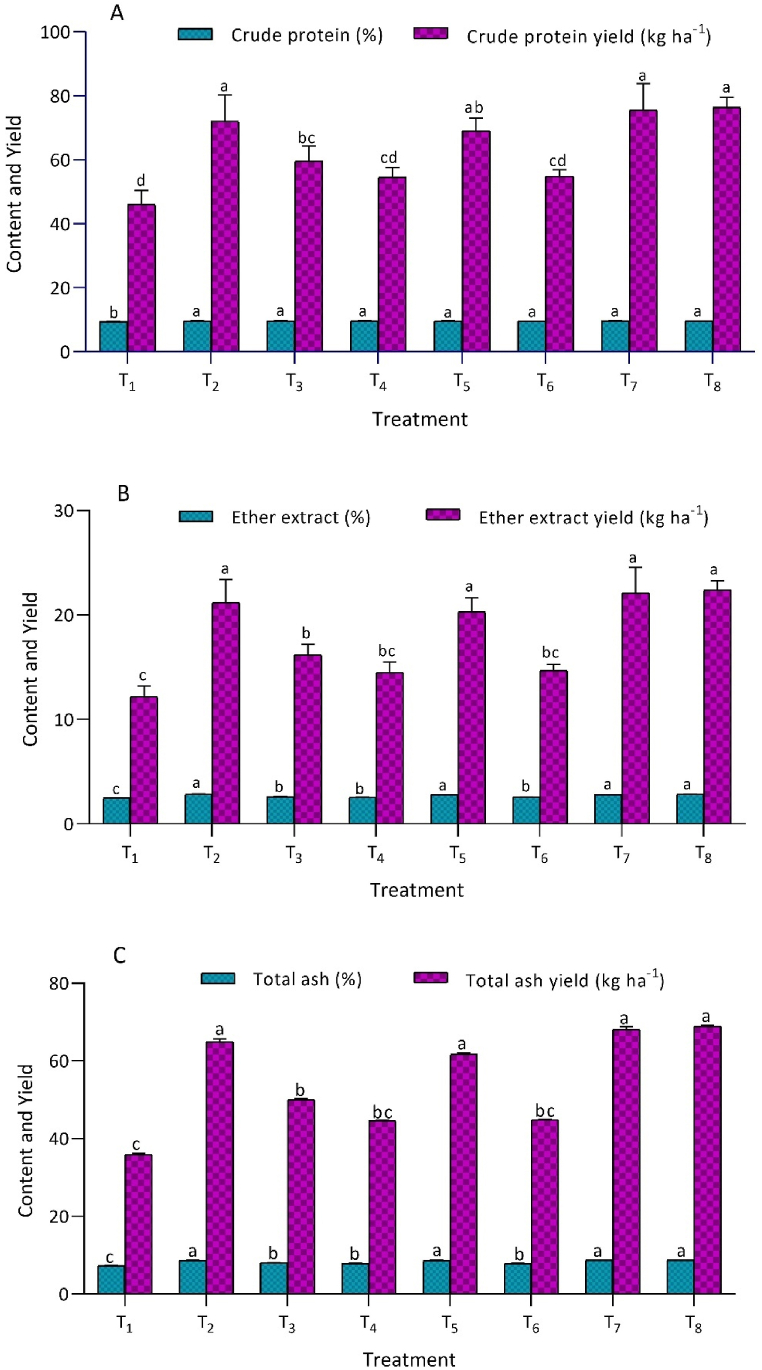
T_1_ - control (No K); T_2_ - recommended dose of potassium fertilizer (RDK) via muriate of potash (MOP); T_3_ - 75 % RDK (MOP) + nano K fertilizer spray at 25 and 40 days after sowing (DAS); T_4_ - 50 % RDK + PGPR + nano K fertilizer spray at 25 and 40 DAS; T_5_ - 75 % RDK + PGPR + nano K fertilizer spray at 25 and 40 DAS; T_6_ - 50 % RDK+25 % K infusion through FYM + PGPR + nano K fertilizer spray at 25 and 40 DAS; T_7_ - 75 % RDK + 25 % K enrichment through FYM + PGPR + nano K fertilizer spray at 25 and 40 DAS; T_8_ - 100 % RDK + 25 % K augmentation through FYM + PGPR and nano K fertilizer spray at 25 and 40 DAS. Fig. 2. Effect of integrated potassium management on proximate composition of Chinese cabbage: A) crude protein content and crude protein yield, B) ether extract content and ether extract yield, and C) total ash content and total ash yield.

Crude protein percentage and crude protein yield exhibited a statistically significant (*p* ≤ 0.05) response to integrated K fertilization (Fig. 2A). Treatments applied with K showed significant superiority over the control (T_1_). Specifically, treatment T_8_, resulted in the highest crude protein content of 9.71 ± 0.01 % and a crude protein yield of 76.4 ± 3.21 kg ha^−1^. This treatment showed statistical similarity with T_7_, which had a crude protein content of 9.69 ± 0.06 % and a crude protein yield of 75.5 ± 8.26 kg ha^−1^. Additionally, T_2_ exhibited a crude protein content of 9.66 ± 0.01 % and a crude protein yield of 72.0 ± 8.25 kg ha^−1^. Similarly, T_5_ had a crude protein content of 9.66 ± 0.05 % and a crude protein yield of 69.0 ± 4.07 kg ha^−1^. Both T_2_ and T_5_ were significantly superior to the control (T_1_), which recorded crude protein content of 9.43 ± 0.10 % and crude protein yield of 46.0 ± 4.46 kg ha^−1^. The study highlights the substantial role of integrated potassium management in enhancing crude protein and crude protein yield in Chinese cabbage.

The total ash content and yield of Chinese cabbage were significantly influenced by integrated potassium application across different treatments (Fig. 2B). Treatment T_8_ had the highest ash content (8.75 ± 0.01 %) and ash yield (68.9 ± 2.88 kg ha^−1^), comparable to T_7_ (8.74 ± 0.02 % and 68.1 ± 7.39 kg ha^−1^). Treatments T_2_ (8.70 ± 0.18 % and 64.9 ± 8.61 kg ha^−1^) and T_5_ (8.64 ± 0.27 % and 61.7 ± 2.80 kg ha^−1^) also showed high ash content and yield. The lowest values were in T_1_ (7.35 ± 0.02 % and 35.9 ± 3.15 kg ha^−1^). Statistical analysis confirmed the significant impact of integrated potassium management on both ash content and yield in Chinese cabbage.

Integrated K application significantly enhances both ether extract content and ether extract yield (Fig. 2C). Specifically, treatment T_8_ demonstrated the highest ether extract content (2.85 ± 0.01 %) and yield (22.4 ± 0.88 kg ha^−1^). Treatment T_7_ followed closely with an ether extract content of 2.84 ± 0.01 % and a yield of 22.1 ± 2.45 kg ha^−1^. Treatment T_2_ exhibited an ether extract content of 2.85 ± 0.05 % and a yield of 21.2 ± 2.19 kg ha^−1^. Treatment T_5_ recorded an ether extract content of 2.84 ± 0.01 % and a yield of 20.3 ± 1.34 kg ha^−1^, significantly outperforming the control treatment, which had an ether extract content of 2.50 ± 0.02 % and a yield of 12.2 ± 1.02 kg ha^−1^. These results highlight the substantial role of integrated K management in augmenting both ether extract content and yield in Chinese cabbage.

### 3.3 Fiber and carbohydrate fractions

Fiber fractions, including NDF, ADF, ADL, and AIA, exhibited significant alterations with integrated K management in Chinese cabbage (Table 2). Notably, all treatments involving integrated K management demonstrated reduced levels of NDF, ADF, ADL, and AIA in comparison to the control (T_1_). Treatment T_8_ yielded significantly lower values (*p* ≤ 0.05) for NDF (53.3 ± 1.71 %), ADF (31.5 ± 0.82 %), ADL (5.96 ± 0.02 %), and AIA (4.29 ± 0.05 %). Notably, these values were statistically comparable to those obtained with treatment T_7_, T_2_, and T_5_, all of which were found to be significantly superior to the control treatment (T_1_). The observed alterations in fiber fractions in Chinese cabbage fodder were attributed to the significant influence of integrated K management strategies employed in the experiment.

**Table 2:** Effect of integrated potassium management on the fiber fraction of Chinese cabbage.

Treatment	NDF (%)	ADF (%)	ADL (%)	AIA (%)	NDIN (%)	NDICP (%)		ADIN (%)	ADICP (%)	
						DM (%)	CP (%)		DM (%)	CP (%)
T_1_	59.0 ± 0.33a	35.2 ± 0.29a	6.64 ± 0.04a	4.88 ± 0.09a	0.580 ± 0.011a	3.62 ± 0.07a	38.4 ± 1.0a	0.289 ± 0.002a	1.81 ± 0.012a	19.2^A^ ± 0.15a
T_2_	53.7 ± 0.00e	31.4 ± 0.86c	6.02 ± 0.04c	4.28 ± 0.02d	0.382 ± 0.030d	2.39 ± 0.19d	24.7 ± 2.0d	0.211 ± 0.002d	1.32 ± 0.012d	13.6^D^±0.12d
T_3_	55.8 ± 0.29cd	33.2 ± 0.04b	6.33 ± 0.17b	4.50 ± 0.09c	0.485 ± 0.008c	3.03 ± 0.05c	31.5 ± 0.8c	0.242 ± 0.002c	1.51 ± 0.012c	15.7^C^ ± 0.15c
T_4_	57.8 ± 0.27ab	34.2 ± 0.53ab	6.50 ± 0.08a	4.75 ± 0.05ab	0.527 ± 0.003b	3.30 ± 0.02b	34.1 ± 0.4b	0.260 ± 0.002b	1.63 ± 0.012b	16.8^B^ ± 0.06b
T_5_	54.2 ± 1.55de	32.0 ± 0.13c	6.04 ± 0.05c	4.36 ± 0.09d	0.379 ± 0.012d	2.37 ± 0.07d	24.5 ± 0.7d	0.213 ± 0.002d	1.33 ± 0.014d	13.8 ± 0.16d
T_6_	57.1 ± 0.77bc	34.3 ± 0.17ab	6.48 ± 0.02ab	4.68 ± 0.02b	0.519 ± 0.015b	3.24 ± 0.09b	33.7 ± 1.0b	0.262 ± 0.001b	1.64 ± 0.005b	17.0^B^ ± 0.04b
T_7_	53.5 ± 0.41e	31.8 ± 0.69c	5.97 ± 0.03c	4.33 ± 0.05d	0.379 ± 0.012d	2.38 ± 0.04d	24.5 ± 0.5d	0.213 ± 0.001d	1.33 ± 0.005d	13.8^D^±0.09d
T_8_	53.3 ± 1.71e	31.5 ± 0.82c	5.96 ± 0.02c	4.29 ± 0.05d	0.370 ± 0.010d	2.31 ± 0.06d	23.8 ± 0.7d	0.210 ± 0.009d	1.31 ± 0.054d	13.5^D^±0.56d
LSD (*p* ≤ 0.05)	1.8	1.2	0.2	0.14	0.027	0.17	2.1	0.008	0.05	0.45

*NDF neutral detergent fibre, *ADF- Acid detergent fibre, *ADL-acid detergent lignin, *AIA – acid insoluble ash, *NDIN – neutral detergent insoluble nitrogen, NDICP- neutral detergent insoluble crude protein, ADIN- acid detergent insoluble nitrogen, ADICP – acid detergent insoluble crude protein.

T_1_ - control (No K); T_2_ - recommended dose of potassium fertilizer (RDK) via muriate of potash (MOP); T_3_ - 75 % RDK (MOP) + nano K fertilizer spray at 25 and 40 days after sowing (DAS); T_4_ - 50 % RDK + PGPR + nano K fertilizer spray at 25 and 40 DAS; T_5_ - 75 % RDK + PGPR + nano K fertilizer spray at 25 and 40 DAS; T_6_ - 50 % RDK+25 % K infusion through FYM + PGPR + nano K fertilizer spray at 25 and 40 DAS; T_7_ - 75 % RDK + 25 % K enrichment through FYM + PGPR + nano K fertilizer spray at 25 and 40 DAS; T_8_ - 100 % RDK + 25 % K augmentation through FYM + PGPR and nano K fertilizer spray at 25 and 40 DAS; DE:digestible energy; DFE: digestible feed energy; ME: metabolizable energy, NE: net energy.

The lowest NDIN value (0.370 ± 0.010) was observed in treatment T_8_, which was statistically similar to T_7_ and T_2_. The highest NDIN value (0.580 ± 0.011) was found in the control treatment (T_1_). For NDICP, T_8_ had the lowest values (2.31 ± 0.06 on a dry matter basis and 23.8 ± 0.7 on a crude protein basis), similar to T_7_, T_2_, and T_5_, while T_1_ had the highest values (3.62 ± 0.07 and 38.4 ± 1.0, respectively). Integrated K management significantly influenced NDIN and NDICP values. Further, treatment T_8_ recorded the lowest ADIN value (0.210 ± 0.009), statistically similar to T_7_, and lower than T_1_ (Table 2). Likewise, the lowest ADICP values (Table 2) were in T_8_ (1.31 ± 0.054 and 13.5 ± 0.56), comparable to T_7_, T_2_, and T_5_, while the highest values were in T_1_ (1.81 ± 0.012 and 19.2 ± 0.15). These results indicate that integrated K fertilization significantly reduces ADIN and ADICP values in Chinese cabbage.

**Fig. 3:**
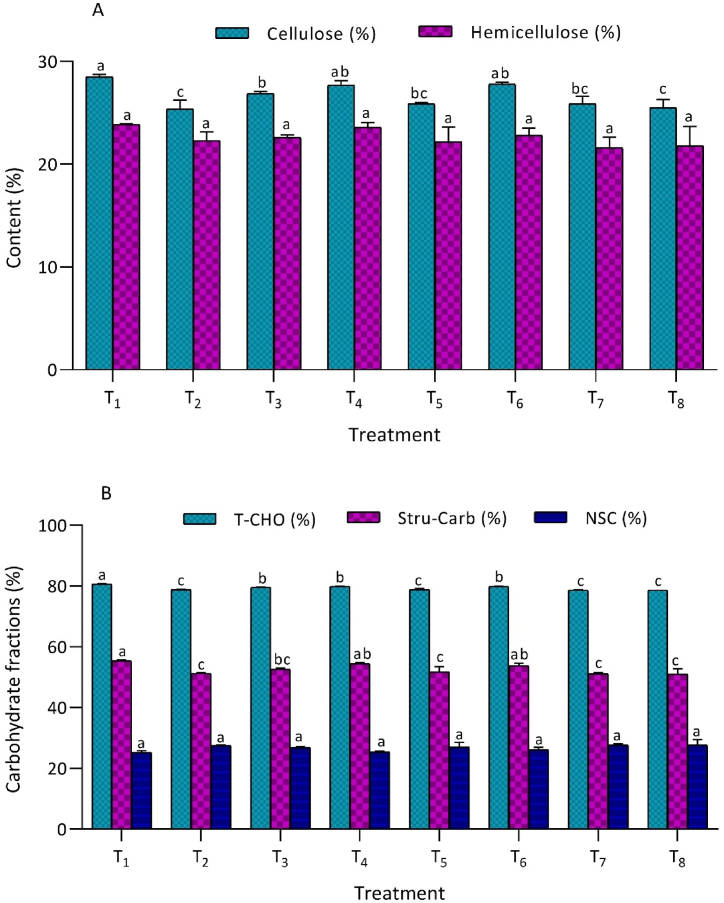
T_1_ - control (No K); T_2_ - recommended dose of potassium fertilizer (RDK) via muriate of potash (MOP); T_3_ - 75 % RDK (MOP) + nano K fertilizer spray at 25 and 40 days after sowing (DAS); T_4_ - 50 % RDK + PGPR + nano K fertilizer spray at 25 and 40 DAS; T_5_ - 75 % RDK + PGPR + nano K fertilizer spray at 25 and 40 DAS; T_6_ - 50 % RDK+25 % K infusion through FYM + PGPR + nano K fertilizer spray at 25 and 40 DAS; T_7_ - 75 % RDK + 25 % K enrichment through FYM + PGPR + nano K fertilizer spray at 25 and 40 DAS; T_8_ - 100 % RDK + 25 % K augmentation through FYM + PGPR and nano K fertilizer spray at 25 and 40 DAS. Fig. 3. Effect of integrated potassium management on proximate composition of Chinese cabbage: A) cellulose and hemicellulose content and B) carbohydrate fractions.

Integrated K management significantly influences the cellulose content in Chinese cabbage. The lowest cellulose content (25.4 ± 0.84 %) was achieved in the T_2_ treatment, which was comparable to the T_8_ (25.54 %) and T_7_ treatments. Similarly, the T_5_ treatment exhibited cellulose content similar to T_8_ and T_7_. Conversely, hemicellulose content remained unaffected by integrated potassium management (Fig. 3A). The minimum hemicellulose content was observed in the T_7_ treatment (21.6 ± 1.07 %), followed closely by the T_8_ treatment (21.8 ± 1.85 %). These findings highlight the nuanced impact of integrated K management on cellulose and hemicellulose content in Chinese cabbage. Significant variations in total carbohydrate content of Chinese cabbage were observed due to integrated K management (Fig. 3B). The highest total carbohydrate (T-CHO) content was found in the T_1_ treatment (80.7 ± 0.07 %), which received no potassium. In contrast, the lowest T-CHO content was recorded in the T_8_ treatment (78.7 ± 0.02 %). This level was comparable to the T_7_ (78.7 ± 0.08 %), T2 (78.8 ± 0.12 %), and T_5_ treatments (78.84 %). The highest structural carbohydrate content (55.4 ± 0.38 %) was also observed in the T_1_ treatment, while the lowest (51.0 ± 1.75 %) was in the T_8_ treatment, with T_2_ and T_5_ showing similar results. Non-structural carbohydrate content showed no significant differences (Fig. 3B).

### 3.4 Feed quality

The DMI content of Chinese cabbage was significantly improved due to the application of integrated K sources in different treatments (Table 3). The highest DMI content (2.25 ± 0.07 %) was observed in treatment T_8_. This result was statistically comparable to T_7_ treatment (2.25 ± 0.02 %). Additionally, treatment T_2_ (2.23 ± 0.00 %) and T_5_ demonstrated substantial DMI content. Conversely, the lowest DMI content was recorded in T_1_ (2.03 ± 0.01 %). Analysis of variance revealed a significant impact of integrated K management on both the green and dry fodder yield of Chinese cabbage.

**Table 3:** Effect of integrated potassium management on the feed quality of Chinese cabbage.

Treatment	DMI (%)	DMD (%)	TDN (%)	RFV	RFQ
T_1_	2.03 ± 0.01c	61.5 ± 0.2c	56.0 ± 0.37c	97.0 ± 0.89d	92.6 ± 1.1d
T_2_	2.23 ± 0.00a	64.4 ± 0.7a	60.8 ± 1.11a	111.6 ± 1.16a	110.5 ± 2.0a
T_3_	2.15 ± 0.01b	63.0 ± 0.7b	58.5 ± 0.05b	105.2 ± 0.59b	102.3 ± 0.6b
T_4_	2.08 ± 0.01c	62.3 ± 0.4bc	57.3 ± 0.69bc	100.3 ± 0.97cd	96.7 ± 1.4cd
T_5_	2.22 ± 0.06ab	64.0 ± 0.1a	60.1 ± 0.17a	109.9 ± 3.31a	108.2 ± 3.4a
T_6_	2.10 ± 0.03bc	62.2 ± 0.1bc	57.1 ± 0.22bc	101.4 ± 1.48bc	97.6 ± 1.5c
T_7_	2.25 ± 0.02a	64.1 ± 0.5a	60.3 ± 0.90a	111.5 ± 0.47a	110.0 ± 1.0a
T_8_	2.25 ± 0.07a	64.4 ± 0.6a	60.7 ± 1.05a	112.4 ± 3.78a	111.2 ± 4.1a
LSD (*p* ≤ 0.05)	0.07	0.9	1.5	3.87	4.4

* DMI- dry matter intake, DMD- Dry matter digestibility, TDN- total digestible nutrient, RFV- relative feed value and RFQ-relative feed quality.

T_1_ - control (No K); T_2_ - recommended dose of potassium fertilizer (RDK) via muriate of potash (MOP); T_3_ - 75 % RDK (MOP) + nano K fertilizer spray at 25 and 40 days after sowing (DAS); T_4_ - 50 % RDK + PGPR + nano K fertilizer spray at 25 and 40 DAS; T_5_ - 75 % RDK + PGPR + nano K fertilizer spray at 25 and 40 DAS; T_6_ - 50 % RDK+25 % K infusion through FYM + PGPR + nano K fertilizer spray at 25 and 40 DAS; T_7_ - 75 % RDK + 25 % K enrichment through FYM + PGPR + nano K fertilizer spray at 25 and 40 DAS; T_8_ - 100 % RDK + 25 % K augmentation through FYM + PGPR and nano K fertilizer spray at 25 and 40 DAS; DE:digestible energy; DFE: digestible feed energy; ME: metabolizable energy, NE: net energy.

The DMD content of Chinese cabbage substantial improved due to application of integrated K management (Table 3). Specifically, treatment T_8_ demonstrated the highest DMD content at 64.4 ± 0.6 %, and found comparable to treatment T_7_, T_2_, and T_5_. The lowest DMD values were observed in treatment T_1_ (control), which recorded values 61.5 ± 0.2 %. Further, the treatment T_2_, exhibited the highest TDN content at 60.7 ± 1.05 % (Table 3). This observation was statistically comparable to the TDN content in treatment T_8_, T_7_, and T_5_. Notably, these treatments significantly outperformed treatment T_1_ (56.0 ± 0.37 %).

The data analysis unveiled that the treatment T_8_ exhibited the highest RFV and RFQ values, recording 112.4 ± 3.78 %, (Table 3). This outcome was statistically comparable to the relative feed values observed in treatment T_7_, T_2_, and T_5_. Notably, treatment T_8_ demonstrated a significant superiority over treatment T_1_ (97.0 ± 0.89 %). Furthermore, treatment T_8_ recorded a notably higher relative feed quality of 111.2 ± 4.1 %, as detailed in Table 3. This result was statistically comparable to the relative feed quality observed in treatment T_7_, T_2_, and T_5_. Importantly, treatment T_8_ demonstrated a significant superiority over treatment T_1_ (92.6 ± 1.1 %).

### 3.5 Overall impact of integrated K management on yield, proximate composition, fiber fractions and feed quality of Chinese cabbage

The impact of integrated K management applied with MOP, PGPR, FYM, and foliar spray of nano potash on yield, proximate composition, fiber fractions and feed quality of Chinese cabbage were analyzed by principal component analysis and correlation matrix among the treatments and various parameters. The data analysis yielded significant principal components, PC1 and PC2, in the experimentation, explaining 99.63 % and 0.36 % variance, respectively (Fig. 4A). Treatments grouped into two clusters: cluster I includes treatments T_1_, T_3_, T_4_, T_6_, and cluster II includes treatments T_2_, T_5_, T_7_, T_8_. Cluster I correlated positively with PC1 but negatively with PC2, while cluster II correlated positively with PC1 and PC2. Parameters grouped into four clusters: cluster I (NDF, T-CHO, Stru-Carb and DMD), cluster II (GFY, TDN, RFV, and RFQ), cluster III (DFY, TA, EE, CP, ADL, AIA NSC, and DMI), and cluster IV (ADF, cellulose, and hemicellulose). Additionally, treatment T_8_ also contributed significantly in improving green fodder yield, total digestible nutrients, structural carbohydrates, total carbohydrates, dry matter digestibility, relative feed value and relative feed quality, and neutral detergent fiber content.

**Fig. 4A:**
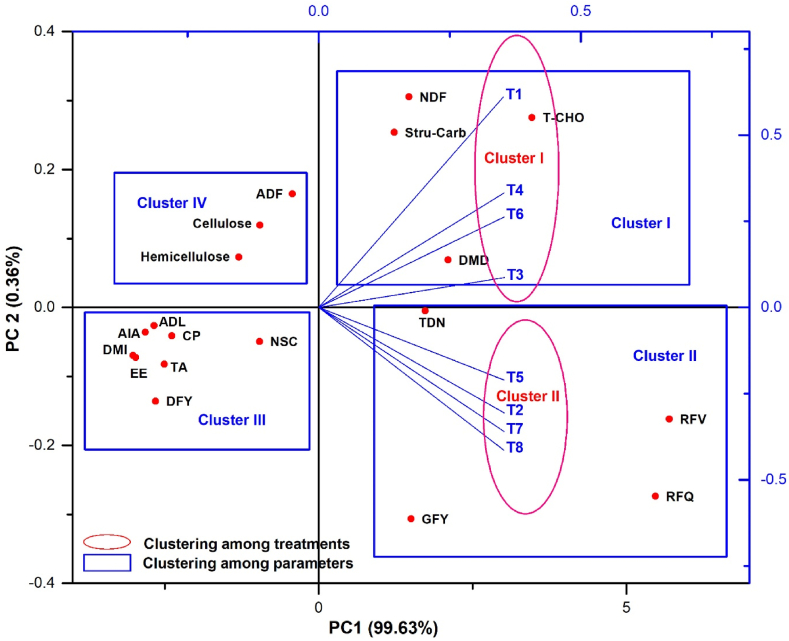
T_1_ - control (No K); T_2_ - recommended dose of potassium fertilizer (RDK) via muriate of potash (MOP); T_3_ - 75 % RDK (MOP) + nano K fertilizer spray at 25 and 40 days after sowing (DAS); T_4_ - 50 % RDK + PGPR + nano K fertilizer spray at 25 and 40 DAS; T_5_ - 75 % RDK + PGPR + nano K fertilizer spray at 25 and 40 DAS; T_6_ - 50 % RDK+25 % K infusion through FYM + PGPR + nano K fertilizer spray at 25 and 40 DAS; T_7_ - 75 % RDK + 25 % K enrichment through FYM + PGPR + nano K fertilizer spray at 25 and 40 DAS; T_8_ - 100 % RDK + 25 % K augmentation through FYM + PGPR and nano K fertilizer spray at 25 and 40 DAS. Fig. 4. Effect of integrated potassium management on (A) principal component analysis and (B) correlation plot matrix among yield, proximate composition and fibre fractions of Chinese cabbage.

The statistical analyzed data shown in the correlation (*r*) plot matrices (Fig. 4B) underscored significant positive associations (*r*) > 0.648 among yield, crude protein, ether extract and total ash content in Chinese cabbage whereas a strong negative correlation (*r*) > −0.693 was observed among yield, crude protein, ether extract, total ash content and fiber fractions such as NDF, ADF, ADL, and AIA in Chinese cabbage.

**Fig. 4B:**
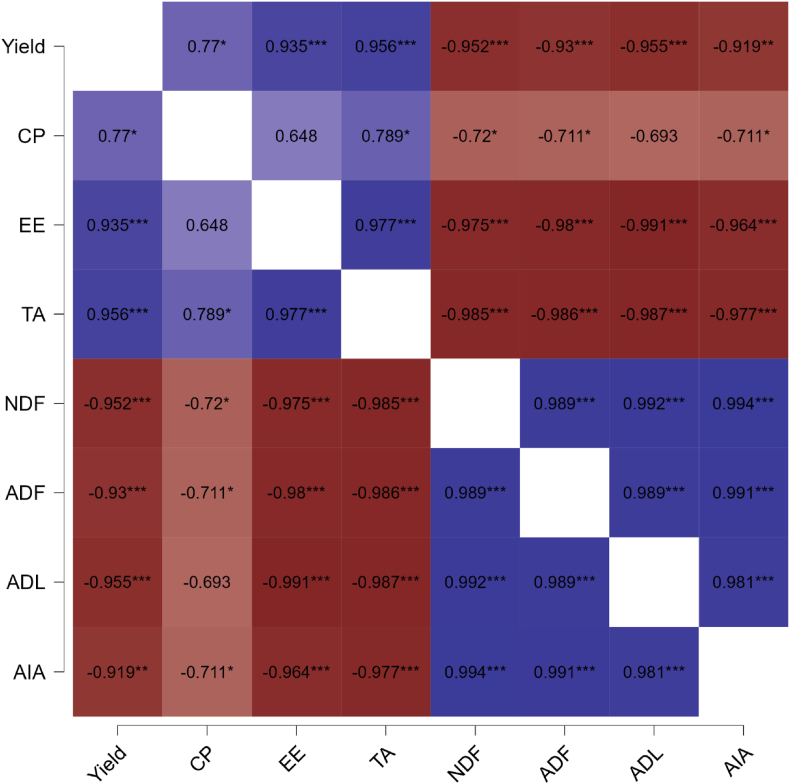

## 4 Discussion

In the current experiment, K fertilization yielded significantly positive results on Chinese cabbage (*Brassica rapa* L. subsp. *chinensis*) fodder yield (Fig. 1). Potassium enhances growth by improving photosynthesis, cell elongation, nutrient translocation, and water absorption in roots [25,26]. Furthermore, K+ is instrumental in regulating auxin concentration and its translocation between roots and shoots [27]. The synergistic effect of plant growth-promoting rhizobacteria, coupled with farmyard manure (FYM), contributed to enhanced fodder yield through ACC-deaminase activity [3,4]. FYM acts as a substrate for soil microbes, promoting increased microbial activity in the rhizosphere, thereby facilitating the enhancement, mobilization, and uptake of nutrients [14]. Kumar et al. [28], have indicated that the application of 60 kg K through MOP and 30 kg K through FYM maximizes yield compared to other treatments. Studies conducted by Iqbal and Umar [29] and Farnia and Ghorbani [30] have demonstrated that nano potash, when combined with biofertilizer, enhances biomass yield. In the present study, the higher green and dry fodder yield of Chinese cabbage (*Brassica rapa* L. subsp. *chinensis*) was attributed to the integrated application of MOP, PGPR, FYM, and nano K spray at 25 and 40 DAS. Similar positive outcomes were also reported by Baljeet et al. [31].

Crude protein content and yield are pivotal indicators for assessing the quality of fodder crops (Fig. 2A). The quantification of crude protein is intricately linked to the availability of nitrogen and its assimilation rate within plants [32]. In the present study, diverse K sources demonstrated a notable influence on nitrogen availability in plants, showcasing a synergistic effect between K and nitrogen. Aulakh and Malhi [33], asserted that K exhibits a synergistic effect on nitrogen uptake and enhances the assimilation rate of various nutrients. Correspondingly, Kumar et al. [28] reported that integrated K application (MOP + FYM) increased crude protein content and yield in maize and wheat crops within a maize-wheat cropping system. The efficacy of foliar application of nano potassium in improving nitrogen content, crude protein content, and crude protein yield in groundnut crops was affirmed by Afify et al. [16]. Additionally, Nosheen et al. [34] highlighted the significant role of plant growth-promoting rhizobacteria (PGPR) in facilitating nitrogen availability and acquisition in canola plants, thereby contributing to an augmentation in crude protein content.

The present investigation highlights the substantive role of diverse K sources in augmenting the availability of various mineral nutrients, thereby contributing to heightened enzymatic activities. This elevation in enzymatic activities, correlates with an increase in ether extract content (Fig. 2B). The observed significant variations in ether extract yield can be attributed to the concomitant higher dry fodder yield and enhanced enzymatic activities facilitated by elevated K availability in the Chinese cabbage under the specific treatment. This finding aligns with previous studies conducted by Kushwaha and Masood [35] and Tiwari et al. [36].

Analysis of total ash content in plants provides insights into the inorganic mineral content, excluding nitrogen and sulfur. The marked differences in total ash content observed in Chinese cabbage (*Brassica rapa* L. subsp. *chinensis*) are a consequence of the augmented potassium levels supplied through MOP, FYM, PGPR, and foliar spray of nano potash (Fig. 2C). Potassium's pivotal role in the translocation process and its synergistic effect on enhancing macronutrient and micronutrient uptake contribute to elevated nutrient concentrations in various plant parts [37]. The application of MOP augments potassium availability for plant uptake, while the use of PGPR and FYM, both independently and in combination, heightens the soluble nutrient concentration in the plant root zone, thereby facilitating increased nutrient uptake [38,39]. Additionally, nano potash application amplifies potassium accumulation in different plant parts. The variations in total ash yield stem from the concurrent higher dry fodder yield and increased mineral nutrient concentration in Chinese cabbage under the specific treatment, aligning with findings reported by Ayub et al. [1] and Bhakar et al. [2]. Further, the observed improvements in ether extract content and ether extract yield in Chinese cabbage can be attributed to the heightened availability of K, promoting the activation of enzymes responsible for oil content production in plants, as reported by Singh et al. [40].

Fiber fractions constitute pivotal indicators of fodder quality, with their abundance significantly influencing digestibility. The present investigation observed a noteworthy impact on various fiber fractions, namely NDF, ADF, ADL, and AIA, due to the integrated K fertilization sources (Table 2). Baljeet et al. [31], which underscored the efficacy of balanced nutrient application in reducing fiber fractions in crops. It is well-established that nutrient deficiencies can impede plant metabolic activities, growth, and development. Specifically, a K deficiency induces stress conditions, resulting in elevated NDF, ADF, ADL, and AIA content, as noted by Pholsen and Suksri [41] and Balabanli et al. [42].

The observed differences in NDIN percentage and NDICP percentage on a dry matter basis among treatments may be ascribed to the varying NDF content in treatments subject to integrated K fertilization. Likewise, disparities in ADIN and ADICP on a dry matter basis among treatments could be attributed to variations in ADF content in treatments subjected to integrated K fertilization. These outcomes resonate with the findings of previous researchers, Yolcu et al. [43], Yolcu et al. [44], Matsi et al. [45] and Qiu et al. [46].

Cellulose and hemicellulose constitute essential components of the plant cell wall. The levels of cellulose and hemicellulose within plants exhibit a close association with the content of ADF, NDF, and ADL. In the current investigation, the concentrations of ADF, NDF, and ADL were influenced by the availability of potassium through the integrated K application. Treatment T1, characterized by the highest recorded values of ADF, NDF, and ADL, demonstrated elevated cellulose (%) and hemicellulose content (%) (Fig. 3A). These outcomes align closely with the findings of Pholsen and Suksri [41] and Balabanli et al. [42], who posit that the application of a balanced fertilizer dose mitigates fiber fraction within the plant cell wall.

The treatment incorporating integrated K fertilization exhibited superior values in DMI, DMD, TDN, RFV, and RFQ values compared to the control (T_1_) The DMI is known to be inversely proportional to NDF content in plants [47] whereas, the observed variation in DMD among treatments is attributed to the positive association of DMD with crude protein and its negative correlation with NDF, ADF, and ADL content in plants [[48], [49], [50]]. The TDN values displays a negative relationship with ADF and NDF content in plants [51]. Further, RFV is gauged based on intake potential and DMD content of the fodder [52]. The present study establishes a negative correlation between integrated K application and the NDF and ADF content of the feed. These findings align with the studies of Kaithwas et al. [53] and Tokas et al. [54], confirming the consistency of results across different investigations.

The investigation revealed a robust positive correlation through improving yield, proximate composition, feed quality and reduction in fiber and carbohydrate fractions in Chinese cabbage under integrated K management (Fig. 4A). The correlation matrices (Fig. 4B) elucidate noteworthy positive associations, exceeding 0.648, among the variables of yield, crude protein, ether extract, and total ash content within the context of Chinese cabbage. Conversely, a robust negative correlation, surpassing −0.693, is evident among yield, crude protein, ether extract, total ash content, and fiber fractions, specifically NDF, ADF, ADL, and AIA, in Chinese cabbage.

## 5 Conclusion

Integrated K management approach, specifically incorporating 100 % RDK through MOP, 25 % K through FYM, PGPR, and two applications of nano potassium spray, facilitates the attainment of higher fodder quantity in Chinese cabbage (*Brassica rapa* L. subsp. *chinensis*). The integrated application of MOP, PGPR, FYM, and foliar spray of nano potash significantly augments total ash content, ether extract content, and crude protein content. Simultaneously, it markedly diminishes NDF, ADF, ADL, AIA, cellulose (%), hemicellulose (%), and various carbohydrate fractions. Consequently, it is deduced that the integrated application of MOP, FYM, PGPR, and nano potash holds promise for enhancing both the yield and physio-biochemical quality of Chinese cabbage.

## CRediT authorship contribution statement

**Mahendra Choudhary:** Writing – original draft, Methodology, Investigation, Formal analysis, Data curation, Conceptualization. **Sourabh Kumar:** Writing – original draft, Formal analysis, Conceptualization. **Santosh Onte:** Writing – original draft, Conceptualization. **Vijendra Kumar Meena:** Supervision, Data curation. **Dhruba Malakar:** Writing – review & editing, Conceptualization. **Kamal Garg:** Writing – original draft, Formal analysis. **Sanjeev Kumar:** Writing – review & editing, Project administration, Methodology, Formal analysis, Data curation, Conceptualization. **Mahendra Vikram Singh Rajawat:** Writing – review & editing, Conceptualization. **Mukesh Kumar Awasthi:** Writing – review & editing. **Balendu Shekher Giri:** Writing – review & editing, Resources, Data curation. **Durgesh Kumar Jaiswal:** Writing – review & editing, Formal analysis. **Shiva Dhar:** Writing – review & editing, Conceptualization. **Elisa Azura Azman:** Writing – original draft, Formal analysis, Data curation. **Sanjivkumar Angadrao Kochewad:** Writing – review & editing, Formal analysis.

## Declaration of competing interest

Authors declare that there is no conflict of interest.
