# Supplementary figures and images for: Optimizing crop quality and yield: Assessing the impact of integrated potassium management on Chinese cabbage (Brassica rapa L. subsp. chinensis)
Source: Heliyon. 2024 Aug 16;10(17):e36208. doi: 10.1016/j.heliyon.2024.e36208 (PMC11388781; doi:10.1016/j.heliyon.2024.e36208)

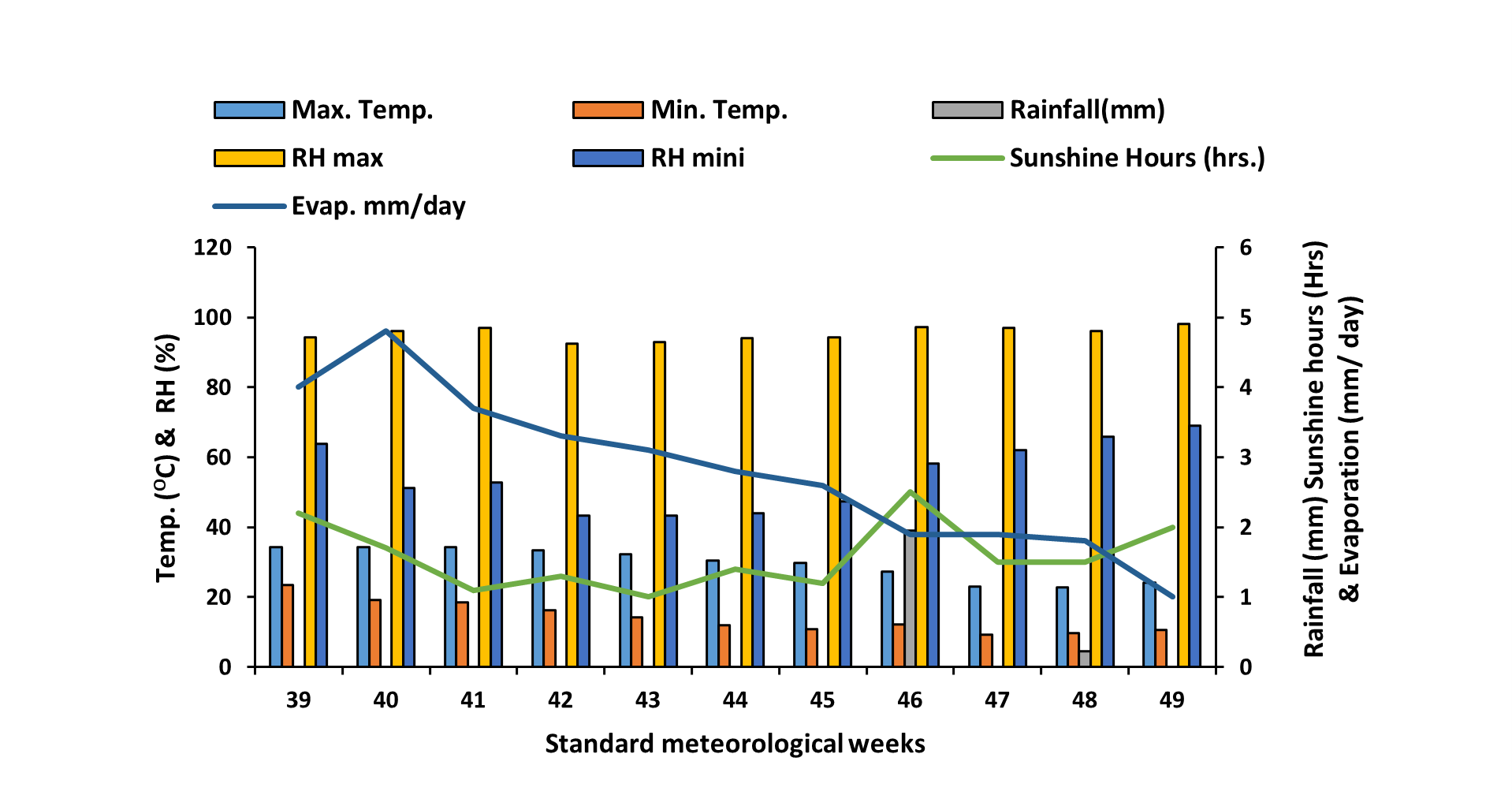


**Suppl. Fig. 1. Weather conditions during the crop growing season in 2020.**

Supplement: Multimedia component 1 [file mmc1.docx]
